# A duplex real-time fluorescent quantitative PCR method for simultaneous and rapid detection of *Actinobacillus pleuropneumoniae* and influenza A virus

**DOI:** 10.3389/fvets.2026.1774637

**Published:** 2026-02-23

**Authors:** Yu-Jie Ren, Hua Lin, Dao-Jian Yu, Jing Zhang, Fei Yang, Bai-Yi Zhang, Ying-Qi Chen, Hai-Xin Long, Li-Li Ren

**Affiliations:** 1 Science and Technology Research Center of China Customs, Beijing, China; 2 Chengdu Customs Technical Center, Chengdu, Sichuan, China; 3 Animal & Plant Inspection and Quarantine Technology Center of Shenzhen Customs, Shenzhen, Guangdong, China; 4 Beijing Coyote Biotech Co., Ltd., Beijing, China

**Keywords:** *Actinobacillus pleuropneumoniae*, duplex real-time fluorescent quantitative PCR, influenza A (H1N1) virus, point-of-care testing, swine respiratory disease

## Abstract

Co-infections involving *Actinobacillus pleuropneumoniae* (APP) and influenza A (H1N1) virus present a serious diagnostic challenge in swine respiratory disease, complicating effective outbreak management and control. This study reports the development and validation of a novel duplex TaqMan real-time PCR assay, optimized for the Coyote Flash10 portable automated detection system, for the simultaneous identification and quantification of both pathogens. The assay employs primers and probes specific to the apxIVA virulence gene of APP and the conserved matrix (M) gene of H1N1, with porcine RNase P as an internal control. Validation using recombinant plasmid standards demonstrated a sensitivity of 1 copy/μL for each target, with strong linear correlation (*R*^2^ > 0.99). Calculated amplification efficiencies (APP: 121.6%; H1N1: 118.6%) were marginally above the typical 90–110% range. However, the assay exhibited robust and consistent quantification without evidence of reaction competition. In a simulated clinical matrix, detection limits corresponded to 10,000-fold and 100-fold dilutions for APP and H1N1, respectively. The method showed excellent repeatability (intra-assay CV <3%) and high specificity, with no cross-reactivity against six other common porcine respiratory pathogens. This rapid, closed-tube assay provides a complete result within one hour and offers a practical, point-of-care-compatible solution for on-farm surveillance and the differential diagnosis of complex respiratory co-infections in swine populations. A key limitation of this study is that validation was performed primarily using simulated samples; future validation with authentic clinical samples will further confirm the method’s clinical utility.

## 1 Introduction

*Actinobacillus pleuropneumoniae* (APP) and influenza A (H1N1) virus are two major types of respiratory pathogens posing a severe threat to the global swine industry and public health security (1–3). APP is a non-motile, encapsulated Gram-negative bacterium primarily transmitted through droplets from chronically or subclinically infected animals (4). Infection with APP can lead to severe fibrinous hemorrhagic and necrotic pleuropneumonia (5), significantly increasing swine mortality and causing billions of dollars in economic losses annually (6). Since the 2009 global pandemic, the H1N1 virus has continuously undergone cross-species transmission between humans and pigs, not only causing respiratory disease and reduced production performance in pigs but also significantly increasing the risk of human infection, presenting an ongoing public health challenge (7, 8).

Real-time fluorescent quantitative PCR (qPCR) technology, known for its high sensitivity, strong specificity, and rapid quantitative capability, has become a mainstream technique for pathogen detection (9). In recent years, point-of-care testing (POCT) fully automated nucleic acid detection analyzers have demonstrated significant value in on-site farm screening, primary veterinary laboratories, and cross-species transmission monitoring due to their portability, automation, and rapid detection features. However, traditional singleplex qPCR requires separate reactions for each target, making the process cumbersome, time-consuming, and resource-intensive, which struggles to meet the need for synchronous detection of multiple pathogens in clinical samples. As a new-generation POCT platform, the Coyote Flash10 fully automated nucleic acid detection analyzer, with its modular design and high-throughput temperature control system, provides the hardware foundation for rapid implementation of multiplex qPCR. Nevertheless, compatible detection methods still require targeted development.

Multiplex qPCR technology enables simultaneous detection of multiple targets within a single reaction tube, significantly improving detection throughput and reducing costs (10). Currently, qPCR detection for APP primarily targets its capsular polysaccharide synthesis gene (*cps*) (11) or toxin genes (e.g., *apx*) (12, 13), while detection for H1N1 virus typically targets conserved genes such as the matrix protein gene (*M*) (14) or the hemagglutinin gene (*HA*) (14, 15). Although standard singleplex qPCR methods for these two pathogens exist, no duplex qPCR detection protocol suitable for POCT platforms has been reported to date. This critical technological gap underscores the novelty and practical relevance of the current study, as POCT-compatible duplex assays are urgently needed to address the diagnostic challenges of co-infections in on-site settings.

Therefore, this study, based on the Flash10 fully automated nucleic acid detection analyzer, aims to establish a duplex qPCR method compatible with this platform to achieve simultaneous and rapid detection of APP and H1N1. By screening specific target genes, optimizing the reaction system, and systematically validating its sensitivity, specificity, and repeatability, a synchronous detection technique applicable to clinical samples was constructed. This method is expected to overcome the limitations of singleplex detection. Combined with the automation advantages of POCT devices, it aims to provide immediate and precise technical support for on-site rapid diagnosis of porcine respiratory mixed infections, epidemiological investigations, and transmission risk assessment, holding significant importance for safeguarding the healthy development of the swine industry and public health security.

## 2 Materials and methods

### 2.1 Design of primer and probe sequences

According to Chapter 3.9.3 of the *Manual of Diagnostic Tests and Vaccines for Terrestrial Animals* (13th edition, 2024) (16), primers and probes targeting the conserved region of the *M* gene of H1N1 virus were designed. With reference to National Standard of the People’s Republic of China: Real-time PCR method for detection of porcine circovirus type 2 (Standard No. GB/T 35901-2018), the porcine *RNase P* gene was selected as the internal control (IC), and specific primers and probes for the conserved region of the *apxIVA* gene of APP were designed using Primer 3 software. The length of all target amplicons was controlled within 150 bp. Probes for APP, H1N1, and the IC were labeled at the 5′ end with FAM, ROX, and CY5 fluorescent reporter groups, respectively, and at the 3′ end with corresponding BHQ1, BHQ2, and BHQ2 quencher groups. All primers and probes were synthesized by Sangon Biotech (Shanghai) Co., Ltd., with specific sequences listed in Table 1.

**Table 1:** Primers and probes for duplex qPCR.

Target pathogen	Target gene	Sequence (5′–3′)	Length (bp)
APP	*apxIVA*	F: GGTTTAGCCGAGAAAATAACG	109
		R: CGAATATTTTCTATTTTATGATCTTGG	
		P: **FAM**-TGAATACCAATTTTGAACCGTGACTTTATCCT-**BHQ1**	
H1N1	*M*	F: AGATGAGTCTTCTAACCGAGGTCG	101
		R_1_: TGCAAAAACATCTTCAAGTCTCTG	
		R_2_: TGCAAAGACACTTTCCAGTCTCTG	
		P: **ROX**-TCAGGCCCCCTCAAAGCCGA-**BHQ2**	
IC	*RNase P*	F: AAGTGCTCGGTGCCTTTAGTG	131
		R: GTCCCATAGACTCACCCTGAAGT	
		P: **CY5**-CCTGGCTCACCTGGACAACCTCAAG-**BHQ2**	

The bold formatting is used to highlight the core functional regions of the primers and probes, including the fluorescent reporter groups and quencher groups labeled at the 5’ and 3’ ends of the probes, to distinguish them from the core nucleotide sequences of primers and probes.

### 2.2 Plasmids and nucleic acid controls

Recombinant plasmids containing the APP *apxIVA* gene, H1N1 *M* gene, and the IC *RNase P* gene were synthesized by Sangon Biotech (Shanghai) Co., Ltd. APP inactivated vaccine was purchased from China Animal Husbandry Industry Co., Ltd. Chengdu Pharmaceutical Equipment Factory. H1N1 nucleic acid control and controls for *Mycoplasma hyopneumoniae*, *Glaesserella parasuis*, *Streptococcus suis*, highly pathogenic porcine reproductive and respiratory syndrome virus, African swine fever virus, porcine circovirus type 2, etc., were purchased from Pudao (Beijing) Standard Technology Co., Ltd.

### 2.3 Reaction system and amplification conditions

To establish a robust duplex qPCR system, a series of screening experiments were conducted to optimize key parameters, including primer/probe concentrations and annealing temperature. Each condition under evaluation was tested in triplicate to assess amplification consistency, and no-template controls (NTCs) were rigorously monitored to exclude non-specific amplification. The final reaction conditions were selected based on the following criteria: (a) the earliest and most stable cycle threshold (Ct) values for both APP and H1N1 targets; (b) absence of amplification signal in all NTC replicates; and (c) matched amplification efficiencies between the two targets. The optimal conditions identified through this screening were used in all subsequent method-validation experiments.

The total reaction volume for the optimized duplex qPCR was 50 μL, containing 35 μL of 2× qPCR Master Mix, 5 μL of primer-probe mix, and 10 μL of template. The 2× qPCR master mix contained reaction buffer, Taq DNA polymerase, dNTPs, reverse transcriptase, and RNase-free ddH₂O. In the primer-probe mix, the final concentrations for APP and H1N1 primers were both 0.5 μM, and their probe final concentrations were 0.4 μM and 0.2 μM, respectively. The final concentrations for the IC primer and probe were both 0.1 μM.

Amplification was performed on the Coyote Flash10 fully automated nucleic acid detection analyzer with the following program: 95 °C pre-denaturation for 1 min; followed by 45 cycles: 95 °C denaturation for 1 s, 65 °C annealing with fluorescence signal acquisition for 5 s, 72 °C extension for 1 s. Fluorescence signal acquisition channels were FAM (APP), ROX (H1N1), and CY5 (IC).

The singleplex qPCR detection system served as the control method for independent detection of APP or H1N1, performed according to the corresponding detection standards (GB/T 27539-2011, NY/T 537–2023). Amplification was conducted on a Thermo Fisher QuantStudio^™^ 1 Plus real-time PCR instrument.

### 2.4 Sensitivity and linearity testing

Recombinant plasmids were serially diluted 10-fold using RNase-free ddH₂O. Each dilution was tested in triplicate using the established duplex qPCR system. Standard curves were plotted with the logarithm of plasmid copy number as the *x*-axis and Ct value as the *y*-axis. The correlation coefficient (*R*^2^) was calculated, and the detection limit was determined as the lowest concentration consistently detected in all three replicates.

### 2.5 Amplification efficiency calculation

Amplification efficiency (*E*) for APP and H1N1 was calculated from the slope of the standard curve generated using serial 10-fold dilutions of recombinant plasmids. The slope (*k*) of each regression line was obtained from the equation:


$$\mathrm{Ct}=k\times \log_{10}(N0)+b$$

Amplification efficiency (*E*) was calculated using the MIQE-recommended formula:


$$E=(10^{\left(-1/k\right)}–1)\times 100\%$$

It should be noted that the conventional acceptable range of amplification efficiency for qPCR is 90–110%, and data beyond this range need to be combined with other indicators such as linearity and repeatability to comprehensively evaluate the reaction performance. All calculations were performed using Microsoft Excel (Microsoft Office 365).

### 2.6 Simulated clinical sample detection sensitivity and repeatability testing

Simulated clinical samples were prepared by diluting APP inactivated vaccine and H1N1 nucleic acid control with porcine negative nasal swab matrix working solution at different dilution levels (H1N1: 10×, 50×, 100× dilution; APP: neat, 10×, 100×, 1,000×, 10,000× dilution). The duplex qPCR method established in this study and the singleplex qPCR method referenced to official detection standards—*National Standard of the People’s Republic of China: Animal Influenza Detection*—*Method of real-time RT-PCR for detection of influenza virus A* (Standard No. GB/T 27539–2011) and *Agricultural Industry Standard of the People’s Republic of China: Diagnostic techniques for porcine contagious pleuropneumonia* (Standard No. NY/T 537-2023)—were performed in parallel. The Ct values, detection limits, and repeatability of these assays were subsequently compared and analyzed.

### 2.7 Specificity evaluation

Nucleic acid controls of *Mycoplasma hyopneumoniae*, *Glaesserella parasuis*, *Streptococcus suis*, highly pathogenic porcine reproductive and respiratory syndrome virus, African swine fever virus, and porcine circovirus type 2 were each mixed with an equal volume of porcine negative nasal swab matrix working solution as templates for duplex qPCR amplification to assess cross-reactivity. All reactions required effective amplification in the IC channel (CY5) to ensure system validity. Simultaneously, standard singleplex qPCR methods were used for parallel verification.

## 3 Results

### 3.1 Design of primers and probes for the duplex qPCR method

**Figure 1:**
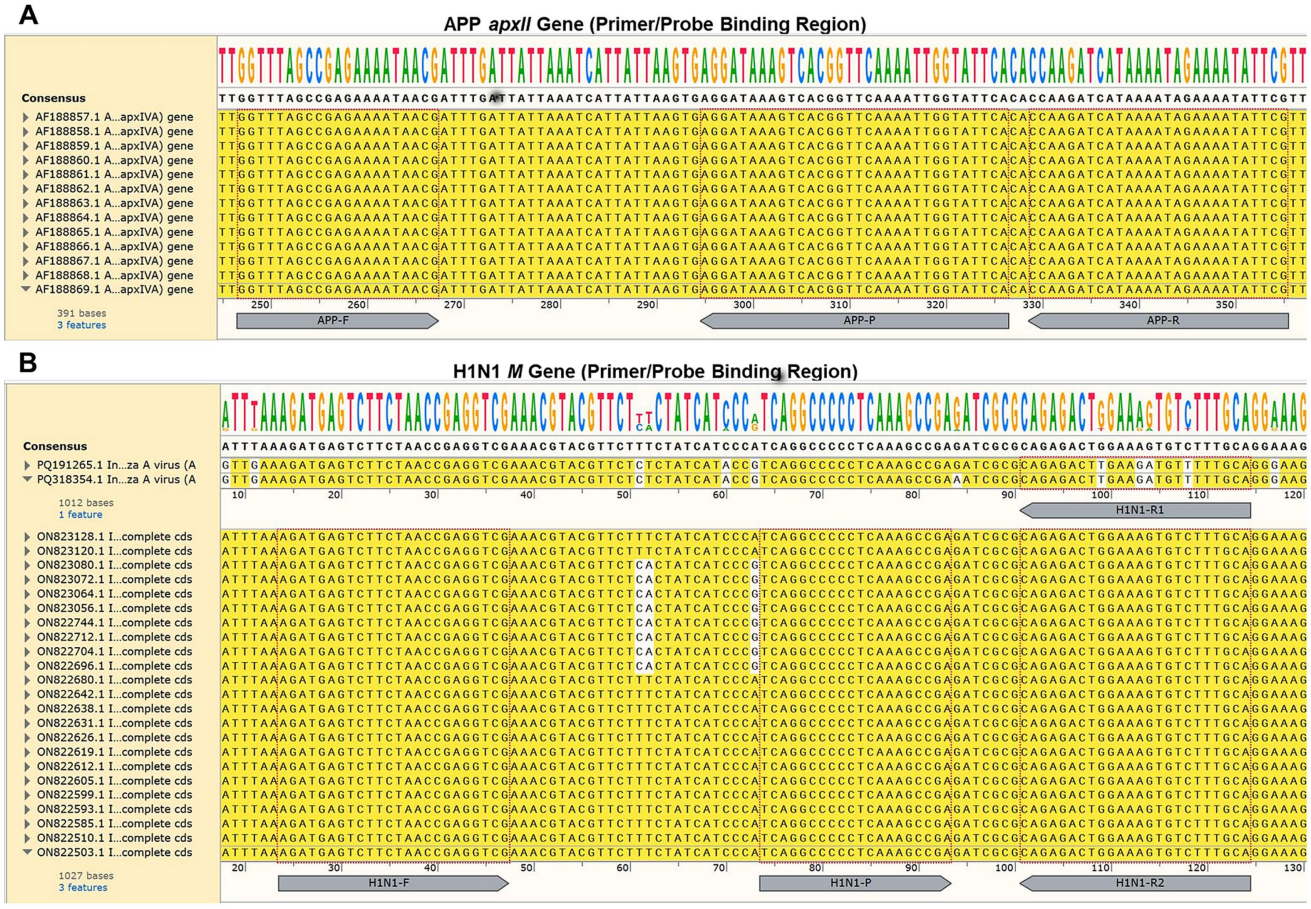
Genomic localization of primer and probe sequences in different APP strains **(A)** and H1N1 isolates **(B)**.

The conserved regions of the *apxII* gene from 13 APP strains (Figure 1A) and the *M* genes from 25 H1N1 isolates (Figure 1B) were aligned separately using the Align Multiple DNA Sequences module in SnapGene software (version 6.0.2). Screenshots of the alignments clearly indicate the precise binding sites of the forward (F) and reverse (R) primers as well as the duplex-labeled TaqMan probe (P) for each target. This design, focused on conserved genomic regions, ensures the amplification of unique pathogen-specific sequences, thereby providing the molecular foundation for simultaneous detection and differentiation of both pathogens in a single reaction tube.

### 3.2 Optimization of the duplex qPCR reaction system

To establish a stable duplex qPCR system, gradient optimization of annealing temperature and primer/probe concentrations was performed in this study. The optimization of annealing temperature was carried out within the range of 55–65 °C, and 65 °C was ultimately determined as the optimal annealing temperature. Under this condition, both the APP (FAM channel) and H1N1 (ROX channel) targets exhibited the earliest Ct values, and the fluorescence signal peaks were significantly higher than those in other temperature groups.

On the basis of this annealing temperature, matrix validation of primer/probe concentrations was conducted. When the final concentrations of both APP and H1N1 primers were set at 0.5 μM, the final concentrations of the probes were 0.4 μM (for APP) and 0.2 μM (for H1N1), respectively, and the final concentration of the IC primer/probe was 0.1 μM, the amplification curves of the two targets showed the highest peaks and the lowest Ct values in their corresponding fluorescence channels. Meanwhile, the negative control showed no fluorescence signal or had a Ct value >40. Therefore, a Ct value <40 was defined as positive, and samples with a Ct value between 35 and 40 were considered suspicious and required retesting.

### 3.3 Sensitivity of the duplex qPCR method and establishment of standard curves

**Figure 2:**
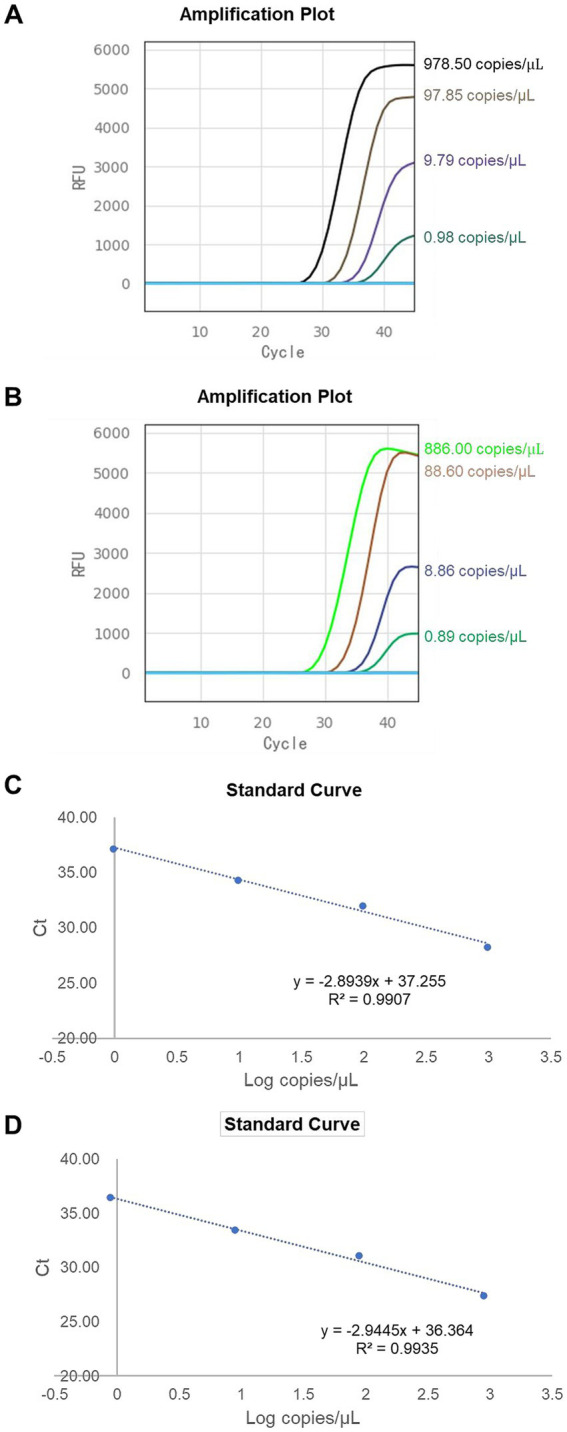
**(A,B)** Amplification curves for APP and H1N1 in the duplex qPCR system using recombinant plasmids at different concentrations. **(C,D)** Standard curves for APP and H1N1 detection by the duplex qPCR system using recombinant plasmids at different concentrations.

The sensitivity of the duplex qPCR method was evaluated using serially diluted recombinant plasmids. Each concentration was analyzed in a minimum of three independent replicates. Meanwhile, the generation of amplification and standard curves was completed following standardized procedures, and the specific process and results are as follows: The amplification plots (Figures 2A,B) presented in this study are direct screenshots of the real-time fluorescence monitoring interface from the Flash10 analyzer’s supporting software. Representative amplification curves from one test are shown in these figures, and the plots depict the fluorescence signal (RFU) versus cycle number for each serial dilution of the recombinant plasmid templates. The curves exhibited good morphology with no abnormal amplification or inhibition, indicating strong reaction specificity. A good negative correlation was observed between template concentration and Ct value, and stable detection was achieved even at concentrations as low as approximately 1 copy/μL (APP 0.98 copies/μL, H1N1 0.89 copies/μL), demonstrating high detection sensitivity of the method. For the construction of standard curves (Figures 2C,D), the mean Ct values obtained from triplicate reactions at each plasmid concentration were plotted against the logarithm of the initial template copy number (log_10_ copies/μL). Linear regression analysis was performed using Microsoft Excel (Microsoft Office 365) to generate the standard curve equation (*y* = *a*log_10_(*x*) + *b*) and to calculate the coefficient of determination (*R*^2^). The *R*^2^ for the linear regression equations of both APP and H1N1 curves was greater than 0.99, indicating a strong linear relationship between Ct values and the logarithm of template concentration.

The slopes of the standard curves for APP and H1N1 were −2.8939 and −2.9445, respectively. Based on these values, the amplification efficiencies were calculated as 121.6% for APP and 118.6% for H1N1, which are slightly higher than the conventional acceptable range of 90–110%. However, combined with the high linearity (*R*^2^ > 0.99) and stable low-concentration detection capability of the method, it can be confirmed that the system still has reliable quantitative performance and good repeatability for the target genes, and there is no obvious competitive inhibition between the two amplification systems.

### 3.4 Detection sensitivity and repeatability of the duplex qPCR method in simulated clinical samples

To evaluate the performance of the detection method in actual complex matrices, the duplex qPCR method from this study and the standard singleplex qPCR method were used to test the same simulated samples at different dilution levels. Results are shown in Table 2.

**Table 2:** Simulated clinical sample detection performance test results.

Target	Method	Dilution factor	Ct values					
			Rep1	Rep2	Rep3	Mean	SD	CV
H1N1	Duplex qPCR (this study)	10×	36.33	36.79	37.36	36.827	0.516	1.40%
		50×	38.49	39.32	38.9	38.903	0.415	1.07%
		100×	38.98	39.3	38.79	39.023	0.258	0.66%
	Singleplex qPCR (standard)	10×	30.927	31.309	31.282	31.173	0.213	0.68%
		50×	33.306	33.335	33.436	33.359	0.068	0.20%
		100×	33.531	34.103	34.354	33.996	0.422	1.24%
APP	Duplex qPCR (this study)	Neat	27.53	28.06	28.08	27.890	0.312	1.12%
		10×	31.18	30.12	31.14	30.813	0.601	1.95%
		100×	31.94	32.82	32.19	32.317	0.453	1.40%
		1,000×	35.1	36.94	35.94	35.993	0.921	2.56%
		10,000×	37.73	38.76	38.09	38.19	0.523	1.37%
	Singleplex qPCR (standard)	Neat	24.415	24.312	24.337	24.355	0.054	0.22%
		10×	27.014	27.165	27.011	27.063	0.088	0.33%
		100×	32.779	32.489	32.928	32.732	0.223	0.68%
		1,000×	36.944	38.031	36.067	37.014	0.984	2.66%

For H1N1 detection, the duplex qPCR method detected Ct values in all three replicates for samples diluted 10–100 times, while no Ct value was detected in the 1,000-fold diluted sample. Thus, the detection limit for H1N1 by the duplex qPCR method was determined to be 100-fold dilution. The standard singleplex qPCR method detected Ct values in all three replicates for samples diluted 10–100 times. For the 1,000-fold dilution tested in triplicate, Ct values were detected in 2 replicates and not in 1, so the detection limit for the standard singleplex qPCR method was provisionally determined to be 100-fold dilution.

For APP detection, the duplex qPCR method detected Ct values in all three replicates for each dilution from 10- to 10,000-fold. Therefore, the detection limit for APP by the duplex qPCR method was provisionally determined to be a 10,000-fold dilution. The standard singleplex qPCR method detected Ct values in all three replicates for samples diluted from neat to 1,000-fold. For the 10,000-fold dilution tested in triplicate, a Ct value was detected in 1 replicate but not in 2, so the detection limit for the standard singleplex qPCR method was provisionally determined to be 1,000-fold dilution.

Regarding repeatability, the coefficient of variation (CV) for Ct values from the duplex qPCR detection of H1N1 and APP simulated clinical samples were no higher than 1.40 and 2.56%, respectively. For the standard singleplex qPCR, the CVs were no higher than 1.24 and 2.66%, respectively. Both methods demonstrated good repeatability (CV <3%).

In summary, the duplex qPCR method from this study and the standard singleplex qPCR method showed consistent detection limits for H1N1, but the duplex qPCR method exhibited higher sensitivity for APP detection. Both methods showed good repeatability (CV <3%) for detecting the two pathogens.

### 3.5 Specificity analysis of the duplex qPCR method

Specificity testing was performed using templates prepared by mixing porcine negative nasal swab matrix working solution 1:1 with nucleic acid controls from 6 common porcine respiratory pathogens: *Mycoplasma hyopneumoniae*, *Glaesserella parasuis*, *Streptococcus suis*, highly pathogenic porcine reproductive and respiratory syndrome virus, African swine fever virus, and porcine circovirus type 2. Results are shown in Table 3. The detection results of the duplex qPCR and the standard singleplex qPCR were consistent. All six non-target porcine respiratory pathogen nucleic acid controls tested negative. In the duplex qPCR system, all non-target pathogens showed no amplification signal in the FAM or ROX channels, while the internal control channel (CY5) showed stable Ct values (23.77–25.14). This indicates good specificity of the method with no cross-reactivity.

**Table 3:** Specificity test results.

Pathogen	Ct values in duplex qPCR system			Ct values in standard singleplex qPCR
	FAM channel	ROX channel	CY5 channel	
*Mycoplasma hyopneumoniae* control	—	—	25.14	—
*Glaesserella parasuis* control	—	—	24.68	—
*Streptococcus sui* control	—	—	24.83	—
Highly pathogenic PRRSV control	—	—	23.77	—
African swine fever virus control	—	—	24.97	—
Porcine circovirus type 2 control	—	—	24.65	—

## 4 Discussion

This study successfully established a duplex qPCR method based on a POCT platform for the synchronous detection of APP and H1N1 virus. Through meticulous design of primers and probes targeting conserved regions of the pathogen genes and systematic optimization of the reaction system, efficient and specific amplification of both pathogens within a single reaction tube was achieved.

The experimental results indicate that the method exhibits high sensitivity, with a detection limit of 1 copy/μL for pure plasmid templates. In simulated clinical samples, its sensitivity for APP detection surpassed that of the national standard-recommended singleplex qPCR method. This may be attributed to the optimized reaction system more effectively mitigating matrix inhibition effects. Good linearity (*R*^2^ > 0.99) and repeatability (CV <3%) demonstrated the method’s reliability and stability. The amplification efficiencies of APP (121.6%) and H1N1 (118.6%) exceeded the conventional acceptable range of 90–110%. Potential causes for this slight elevation may include the synergistic effects of primer-probe concentrations in the duplex system, minor deviations in reaction conditions (e.g., annealing temperature precision), or the absence of target competition allowing for more efficient polymerase activity. Despite this, the efficiencies are close to the ideal 100% threshold and, when combined with high linearity (*R*^2^ > 0.99) and stable low-concentration detection, the system maintains reliable quantitative performance. Importantly, no evidence of cross-inhibition between the two amplification systems was observed, confirming the compatibility of the duplex design. To mitigate potential impacts of elevated efficiency on quantitative accuracy, future iterations could optimize primer concentrations (e.g., slight reduction of APP primer concentration) or adjust annealing temperature by ±0.5–1 °C to fine-tune amplification kinetics. These results align with MIQE guidelines, which emphasize that amplification efficiency should be interpreted alongside other performance metrics rather than considered in isolation. Rigorous specificity validation also ensured its differential diagnostic capability in complex sample backgrounds.

Compared to traditional singleplex detection, this method integrates the detection of two pathogens into one reaction, completing the assay in approximately one hour, significantly saving time, reagents, and sample volume. Furthermore, the method is fully compatible with portable, fully automated nucleic acid detectors like the Flash10, realizing fully automated and closed processing from sample addition to result analysis. This greatly reduces operational complexity and the risk of aerosol contamination, making it suitable for rapid on-site screening scenarios such as farms and border quarantine stations. The innovation of this method holds substantial practical impact for field diagnostics and outbreak management: (1) On pig farms, rapid simultaneous detection enables timely isolation of co-infected animals, reducing transmission risk and minimizing economic losses; (2) In border quarantine and mobile surveillance settings, the POCT compatibility allows for on-site testing of imported/exported swine or samples from high-risk areas, enhancing biosecurity; (3) During outbreaks, the method facilitates rapid etiological confirmation, supporting targeted control measures (e.g., distinguishing bacterial vs. viral contributions to respiratory disease) and preventing overuse of antibiotics. These applications address critical gaps in current disease management, where delayed or sequential testing often hinders effective response.

It is important to acknowledge that the validation of this method was primarily performed using simulated clinical samples, which were constructed by spiking target antigens into porcine negative nasal swab matrices. This simulated matrix was specifically designed to mimic the biological composition (including mucosal components, proteins, and potential inhibitory substances) of authentic clinical specimens commonly encountered in swine respiratory disease diagnostics. The robust performance of the assay in these simulated samples—evidenced by high sensitivity (10,000-fold dilution for APP, 100-fold dilution for H1N1), strong linearity (*R*^2^ > 0.99), excellent repeatability (CV <3%), and resistance to matrix interference—provides compelling evidence for its potential clinical utility. Notably, the assay’s inclusion of an internal control (porcine RNase P gene) further enhances its reliability in complex sample matrices by monitoring sample quality and reaction efficiency, mitigating the impact of potential inhibitors that may be present in authentic clinical samples.

The novelty of this study—being the first to report a POCT-compatible duplex qPCR assay for simultaneous detection of APP and H1N1—remains fully supported by the current data. The method directly addresses the unmet need for rapid, on-site multiplex detection of these co-infecting pathogens, which is a key diagnostic challenge in swine respiratory disease management. While clinical validation with authentic samples would further extend the translational value of the assay, this represents a natural subsequent step for real-world application rather than a requirement for the current study’s core objectives. The systematic preclinical validation completed herein—encompassing sensitivity, specificity, linearity, repeatability, and matrix compatibility—fulfills the essential criteria for evaluating a novel diagnostic assay, demonstrating its readiness for adoption in field or clinical settings with site-specific validation as deemed appropriate by end-users. Future work may include clinical evaluation with authentic samples to generate real-world performance data, but this does not diminish the scientific rigor or practical significance of the current findings.

To further enhance specificity, future work will include additional specificity testing with field isolates and related pathogens if feasible. This will involve testing clinical isolates of APP (serotypes 1–15), H1N1 variants (e.g., 2009 pandemic strain, swine-adapted variants), and closely related pathogens (e.g., *Actinobacillus suis*, influenza A H3N2 virus) to confirm the method’s ability to distinguish target pathogens from genetically similar strains.

## Data Availability

The original contributions presented in the study are included in the article/supplementary material, further inquiries can be directed to the corresponding author.
